# Evidence of cospeciation between termites and their gut bacteria on a geological time scale

**DOI:** 10.1098/rspb.2023.0619

**Published:** 2023-06-28

**Authors:** Jigyasa Arora, Aleš Buček, Simon Hellemans, Tereza Beránková, Johanna Romero Arias, Brian L. Fisher, Crystal Clitheroe, Andreas Brune, Yukihiro Kinjo, Jan Šobotník, Thomas Bourguignon

**Affiliations:** ^1^ Okinawa Institute of Science and Technology Graduate University, 1919-1 Tancha, Onna-son, Okinawa 904-0495, Japan; ^2^ Faculty of Tropical AgriScience, Czech University of Life Sciences, Kamýcká 129, Suchdol, 165 00, Prague 6, Czech Republic; ^3^ Madagascar Biodiversity Center, Parc Botanique et Zoologique de Tsimbazaza, Antananarivo 101, Madagascar; ^4^ California Academy of Sciences, San Francisco, CA, USA; ^5^ Research Group Insect Gut Microbiology and Symbiosis, Max Planck Institute for Terrestrial Microbiology, Marburg, 35043, Germany; ^6^ College of Economics and Environmental Policy, Okinawa International University, 2-6-1 Ginowan, Ginowan, 901-2701, Okinawa, Japan; ^7^ Biology Centre, Czech Academy of Sciences, Ceske Budejovice, 37011, Czech Republic

**Keywords:** cophylogeny, endosymbionts, isoptera, metagenomics, vertical inheritance

## Abstract

Termites host diverse communities of gut microbes, including many bacterial lineages only found in this habitat. The bacteria endemic to termite guts are transmitted via two routes: a vertical route from parent colonies to daughter colonies and a horizontal route between colonies sometimes belonging to different termite species. The relative importance of both transmission routes in shaping the gut microbiota of termites remains unknown. Using bacterial marker genes derived from the gut metagenomes of 197 termites and one *Cryptocercus* cockroach, we show that bacteria endemic to termite guts are mostly transferred vertically. We identified 18 lineages of gut bacteria showing cophylogenetic patterns with termites over tens of millions of years. Horizontal transfer rates estimated for 16 bacterial lineages were within the range of those estimated for 15 mitochondrial genes, suggesting that horizontal transfers are uncommon and vertical transfers are the dominant transmission route in these lineages. Some of these associations probably date back more than 150 million years and are an order of magnitude older than the cophylogenetic patterns between mammalian hosts and their gut bacteria. Our results suggest that termites have cospeciated with their gut bacteria since first appearing in the geological record.

## 1. Introduction

Symbiotic associations with bacteria are pervasive across the animal tree of life [1]. Some of these associations involve partners that have continuously and reciprocally adapted to each other over extended evolutionary time scales, a phenomenon referred to as coevolution [2]. The coevolution between bacteria and their hosts is sometimes coupled with mechanisms of vertical transmission leading to cospeciation and cophylogenetic patterns, whereby the phylogenetic trees of both symbiotic partners show congruence in terms of topology and timing [3,4]. For example, many insects, such as aphids, cockroaches or whiteflies (e.g. [5–7]), and many marine invertebrates, such as vesicomyid clams or catenulid flatworms (e.g. [8,9]), harbour intracellular bacterial endosymbionts with phylogenetic trees closely matching that of their hosts. The congruence between host and symbiont phylogenetic trees reflects the strict vertical transmission of intracellular endosymbionts from mother to eggs or embryos [10,11], which sometimes takes place over several hundred million years [5].

In contrast with maternally inherited intracellular endosymbionts, there are no clear examples of gut bacteria being vertically transmitted on hundred-million-year time scales. Cophylogenetic patterns between gut bacteria and their hosts have been identified primarily in obligate symbioses with highly specialized modes of symbiont transmission, such as the nutritional symbiont *Candidatus* Ishikawaella capsulata of plataspid stinkbugs [12] and the pectinolytic *Candidatus* Stammera of cassidinine leaf beetles [13]. The rarity of cophylogenetic patterns between gut bacteria and their host may be linked to the difficulty of establishing stable vertical transmission routes, especially in species with limited social interactions. In social insects, such as bees, ants and termites, nest-mates experience frequent social contacts and often exchange gut fluid through trophallaxis, a behaviour providing a stable route of gut bacterial transmission across generations [11]. In consequence, some social insects, such as the corbiculate bees, present cophylogenetic patterns with their gut bacteria [14,15], indicating that sociality may lead to the coevolution of gut bacteria with their host at geological time scales.

Cophylogenetic analyses have rarely been performed for animals with complex bacterial gut microbiota, perhaps because many studies have relied upon 16S rRNA sequences, a marker that diverges at about 1% per 50 Myr [16]. Because of its slow rate of evolution, the 16S rRNA gene does not provide the taxonomic resolution required to resolve cophylogenetic patterns. Studies of the gut microbiota of humans and great apes successfully used protein-coding sequences to identify cophylogenetic patterns between several bacterial lineages and their mammalian hosts, suggesting that both partners have coevolved over the past 15 Myr [17,18]. This coevolution may have occurred because of the limited ability of symbionts to survive outside their host as they lost genes involved in key metabolic functions and developed specific oxygen and temperature requirements [18–20]. Here, we used protein-coding marker genes obtained from termite gut metagenomes to analyse the cophylogenetic patterns between termites and their gut bacteria.

Termites host unique gut microbial communities composed of bacteria, archaea and cellulolytic flagellates [21]. The gut flagellates have cospeciated with their hosts since their acquisition by the common ancestor of termites and their sister group, the wood-feeding roach *Cryptocercus* [22–24]. Numerous bacterial lineages occur ubiquitously in all termite species investigated but have never been found outside of termite guts [25,26]. These endemic bacteria are believed to be acquired via two transmission routes: vertical and horizontal [26]. The vertical route involves both colony founders (the king and the queen) and nest-mates that provide each other gut fluid through trophallaxis, ensuring the transmission of gut bacteria among family members and, ultimately, from parent colonies to daughter colonies [27]. The horizontal route involves the transfer of bacteria between unrelated colonies sometimes belonging to different termite species, or the acquisition of environmental bacteria. The relative importance of the vertical and horizontal transmission routes in shaping the gut microbiota of termites remains unknown.

In this study, we searched for evidence of cophylogeny between termites and their gut bacteria. We compared two phylogenetic trees of termites reconstructed using mitochondrial genomes and ultraconserved elements (UCEs), respectively, with phylogenetic trees of gut bacteria reconstructed using 10 independent universally occurring protein-coding marker genes [28]. Our study reveals that horizontal transfers between termite species may not be needed to explain the cophylogenetic patterns between termites and some of their endemic gut bacteria. Our results suggest that some bacterial lineages found in termite guts have been vertically transmitted over the past 150 Myr of termite evolution [29,30].

## 2. Material and methods

### (a) Sample collection and metagenome analyses

We used the gut metagenomes of 141 termite samples and one sample of the cockroach *Cryptocercus kyebangensis* sequenced by Arora *et al*. [31] (electronic supplementary material, table S1). In addition, we sequenced 56 termite gut metagenomes for this study. All samples were preserved in RNA-later, stored for up to several weeks at room temperature, and subsequently stored at −80°C until DNA extraction. We extracted and sequenced DNA and assembled the metagenomes as described in Arora *et al*. [31].

Ten single-copy protein-coding marker genes were extracted from the assemblies using the mOTU software [28,32,33]. Genomes and metagenome-assembled genomes available in the Genome Taxonomy Database GTDB v. 95 [34] were downloaded, and the same 10 single-copy marker genes were extracted as described above.

The taxonomic annotation of the 10 marker genes extracted from termite gut metagenome assemblies was performed using the lowest common ancestor algorithm implemented in DIAMOND BLASTP [35] with e-value ≤ 1e-24 as a threshold. The BLASTP search was performed against the GTDB database v.95 [34]. For downloaded genomes, we used the taxonomic annotation available from GTDB v. 95. The marker gene sequences from termite gut metagenomes and the GTDB database were analysed separately for every phylum. We reconstructed the phylogenetic tree of every phylum comprising more than 10 sequences.

### (b) Reconstruction of marker gene phylogenetic trees

Sequences shorter than half the mean length of the marker gene were removed to improve the accuracy of phylogenetic reconstructions [36,37]. Protein sequences were aligned using MAFFT v. 7.305 with the *-auto* option [38]. Protein alignments were back-translated into their corresponding nucleotide alignments using PAL2NAL [39]. Aligned nucleotide sequences were converted into purines (R) and pyrimidines (Y) using BMGE v. 1.12 [40] to account for the variability of GC content observed across bacterial sequences. Maximum-likelihood (ML) phylogenetic trees were generated using these RY-recoded sequence alignments with IQ-TREE v. 1.6.12 [41]. We used the GTR2 + G + I model of binary state substitution. Node supports were assessed using the ultrafast bootstrap method [42] with the command *-bb 2000* for 2000 bootstrap replicates. The phylogenetic trees of every phylum were rooted using outgroup taxa selected from the bacterial tree of life [34,43]. The phylogenetic trees of archaeal and bacterial clades composed of sequences found exclusively in termite guts and represented by more than 10 termite species were extracted from the phylogenetic trees of each phylum. We refer to these trees, including sequences of termite gut bacteria exclusively, as termite-specific clades (TSCs). This procedure was followed for each marker gene.

### (c) Phylogenetic reconstruction of termites using mitochondrial genomes

We reconstructed the phylogenetic tree of termites using mitochondrial genome sequences. Termite mitochondrial contigs longer than 5000 bp and more than 90% identical to the previously published whole mitochondrial genomes of termites [29,44–49] were identified using BLAST searches [50]. Complete or near-complete mitochondrial genomes were annotated using the MITOS web server [51]. We aligned the 13 protein-coding genes, two ribosomal RNA genes and 22 transfer RNA genes with MAFFT v. 7.305 [38]. All gene alignments were concatenated, and the third codon position of protein-coding genes was removed. The concatenated alignment was divided into four partitions: one for the first codon position of protein-coding genes, one for the second codon position of protein-coding genes, one for the combined transfer RNA genes and one for the combined ribosomal RNA genes. We reconstructed a Bayesian phylogenetic tree using BEAST v. 2.4.8 [52], following the approach described in Arora *et al*. [31]. We used an uncorrelated lognormal relaxed clock as a model of rate variation [53] and a birth–death process as a tree prior [54]. We used the nine fossil calibrations used by Arora *et al*. [31], which we implemented as exponential priors on node times. Sphaerotermitinae and Macrotermitinae were constrained to form a monophyletic group, as supported by phylogenetic trees based on transcriptomes and UCEs [30,55]. Similar constraints were applied to non-Stylotermitidae Neoisoptera, which were constrained to be monophyletic [31].

### (d) Phylogenetic reconstruction of termites using ultraconserved elements

We extracted from each gut metagenome assembly termite UCEs, and their flanking 200 bp at both 5′ and 3′ ends, using PHYLUCE v. 1.6.6 [56] and LASTZ [57]. We used the termite-specific bait set targeting the 50 616 UCE loci described in Hellemans *et al*. [55] and followed the procedure described therein. The UCE dataset produced in this study (Contribution #3 to the Termite UCE Database available at: https://github.com/oist/TER-UCE-DB/) is available on the Dryad Digital Repository (https://doi.org/10.5061/dryad.tmpg4f53w). Loci were aligned with MAFFT [38], as implemented in phyluce_align_seqcap_align. Alignments were trimmed internally using phyluce_align_get_gblocks_trimmed_alignments_from_untrimmed, which implements Gblocks [58,59] with default parameters. UCE loci found in more than 57% of termite gut metagenomes were extracted with phyluce_align_get_only_loci_with_min_taxa. Of those, the 322 loci matching, at least partly, singly annotated exons from the draft genome of *Zootermopsis nevadensis* [60] were used for downstream analyses. The final supermatrix, composed of 322 UCE alignments, was obtained with phyluce_align_format_nexus_files_for_raxml. We carried out ML tree reconstruction on the supermatrix using IQ-TREE v. 1.6.12 with a GTR + G + I model of nucleotide substitution and 1000 ultrafast bootstrap replicates to assess branch supports [41,61].

### (e) Matching termite-specific archaeal and bacterial clades across marker gene trees

The phylogenetic trees reconstructed with the marker gene coding for COG0552 (*ftsY*) were used as references. We attempted to link every TSC found in the phylogenetic trees reconstructed with COG0552 with their counterparts found in the phylogenetic trees reconstructed with the other nine marker genes. To do so, we searched the 198 gut metagenomes for contigs encompassing at least two of the 10 marker genes. The position of each marker gene sequence in their respective phylogenetic trees was used to match TSCs across marker gene trees. We also used the 10 marker genes of the termite gut bacterial genomes found in the GTDB database. Of the 194425 genomes downloaded from the GTDB database, 37 were associated with termite guts.

### (f) Cophylogenetic analyses

We used three approaches to test for cophylogeny between termites and TSCs. For the first approach, we used the R package PACo (Procrustean Approach to Cophylogeny) [62], which implements Procrustean superimposition to estimate the cophylogenetic signal between two phylogenies. The host and symbiont phylogenetic trees were converted into distance matrices using the *cophenetic()* function of the vegan R package [63]. The software was run using the *backtracking* method of randomization that conserves the overall degree of interactions between the two trees [64]. The second approach was the generalized Robinson–Foulds (RF) metric [65]. This method was implemented using the *ClusteringInfoDistance()* function of the TreeDist R package [65]. For the third approach, the host and symbiont phylogenetic trees were matched to find an optimal one-to-one map between branches using the method described by Nye *et al*. [66] and implemented in the *NyeSimilarity()* function of the TreeDist R package [65]. Because the two methods implemented in the TreeDist R package do not allow multiple symbiont tips in one host, each host tip was split into a number of tips of zero branch length equal to the number of archaeal and bacterial symbionts present in the metagenome corresponding to that given tip [67,68]. The strength of the cophylogenetic signal was computed from each cophylogenetic algorithm. Congruence between the host and symbiont trees was determined using 10 000 random permutations. We ran the cophylogenetic analyses on the phylogenetic trees reconstructed with mitochondrial genomes and UCEs.

We estimated the number of host transfer events for each TSC using the GeneRax software [69]. GeneRax is a ML-based method that reconciles the microbial gene tree with the host tree. It estimates rates of horizontal transfers within TSCs, the probability that a microbe is transferred from one host to a random host not ancestral to the donor host. We carried out each cophylogenetic analysis twice, once with the termite phylogenetic tree reconstructed with mitochondrial genomes and once with the tree reconstructed with UCEs. We compared the rates of transfers obtained for TSC trees with the rates of transfers calculated for the 13 protein-coding genes analysed without the third codon position and the two rRNA mitochondrial genes. Because mitochondrial genomes do not recombine, all mitochondrial genes have an identical evolutionary history and experienced no transfer. The positive rates of transfers found for mitochondrial gene trees reflect the uncertainties of phylogenetic reconstructions and provide a baseline for the estimated rates of horizontal transfer values in the absence of horizontal transfer. We consider that the evolutionary history of TSCs is predominantly explained by vertical transfers when their rates of horizontal transfers fall within the range of that found for mitochondrial genes.

## 3. Results and discussion

The sequences were derived from 197 termite gut metagenomes and one *Cryptocercus* metagenome combined with sequences from the GTDB database [34]. Our dataset comprises representatives of all termite families, spanning approximately 150 Myr of evolution, and the main lineages of Termitidae, which arose around 50 Ma [29,30]. It also includes samples of 30 species of *Microcerotermes*, a pantropical termitid genus that appeared around 20 Ma [45]. Therefore, our dataset captured both the intrageneric variations and ancient divergences of the termite hosts.

**Figure 1.**
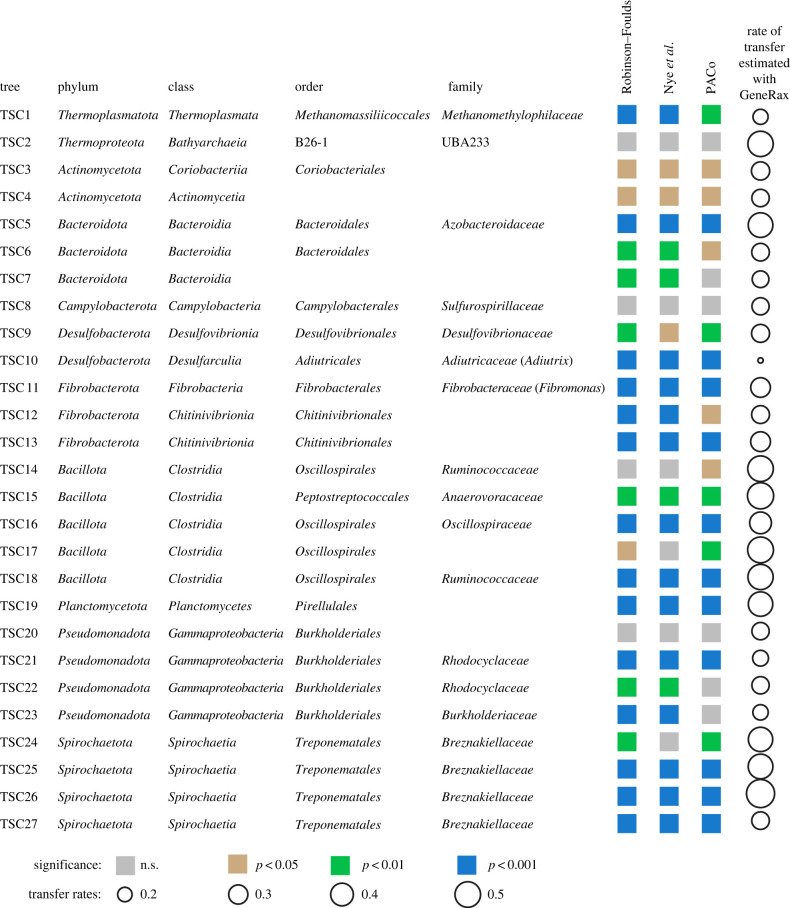
Results of the cophylogenetic analyses performed on the marker gene COG0552 of 27 termite-specific archaeal and bacterial clades (TSCs). The cophylogenetic analyses were performed with three different methods: PACo, the generalized RF metric, and the tree alignment algorithm described by Nye *et al.* [66]. The transfer rates were estimated using the ML method implemented in the GeneRax software.

We reconstructed separate ML phylogenies for each marker gene and each bacterial and archaeal phylum. Then, we searched each tree for TSC composed exclusively of sequences associated with termites and represented in at least 10 termite species. We found between 8 and 34 TSCs per marker gene and selected *ftsY* (COG0552), which was represented by 2299 sequences forming 27 TSCs, as a reference marker gene. The 27 TSCs of COG0552 belonged to nine bacterial and two archaeal phyla. We examined the cophylogenetic signal between each TSC and its termite host using the termite phylogenetic trees reconstructed with mitochondrial and UCE data and three different methods: PACo [62], the generalized RF metric [65], and the tree alignment algorithm described by Nye *et al*. [66]. The results of the analyses performed on the two termite phylogenetic trees were almost identical (electronic supplementary material, table S2), indicating that our analyses were robust to the type of data used to reconstruct the host phylogenetic tree. We discuss the results obtained with the termite mitochondrial phylogenetic tree for simplicity. Eighteen of 27 TSCs showed significant cophylogenetic signals with termites with all three methods (figure 1). We then inferred TSCs from the other nine marker genes and identified their correspondence to the COG0552 marker gene-based TSCs based on the physical linkage of marker genes on contigs (see Material and methods for additional details). Cophylogenetic analyses on corresponding TSCs from all marker genes yielded similar results (electronic supplementary material, table S2), indicating that the choice of reference marker gene did not influence the outcome of our analyses.

The TSCs with the strongest cophylogenetic signals included key components of the gut microbiota of termites. For example, the families *Ruminococcaceae* (phylum *Bacillota*, formerly *Firmicutes*) and *Breznakiellaceae* (phylum *Spirochaetota*), respectively, made up 16.5% and 20.0% of the 16S rRNA gene sequences found in a survey of 94 termite species [26]. *Breznakiellaceae* generally have a fermentative metabolism and include strains capable of reductive acetogenesis [70,71]. They have been isolated from the guts of cockroaches, suggesting that they were already present in the ancestor of termites and their cockroach sister group, Cryptocercidae [71,72]. Therefore, TSCs with essential functions and a long history of association with termites show cophylogenetic signals.

**Figure 2.**
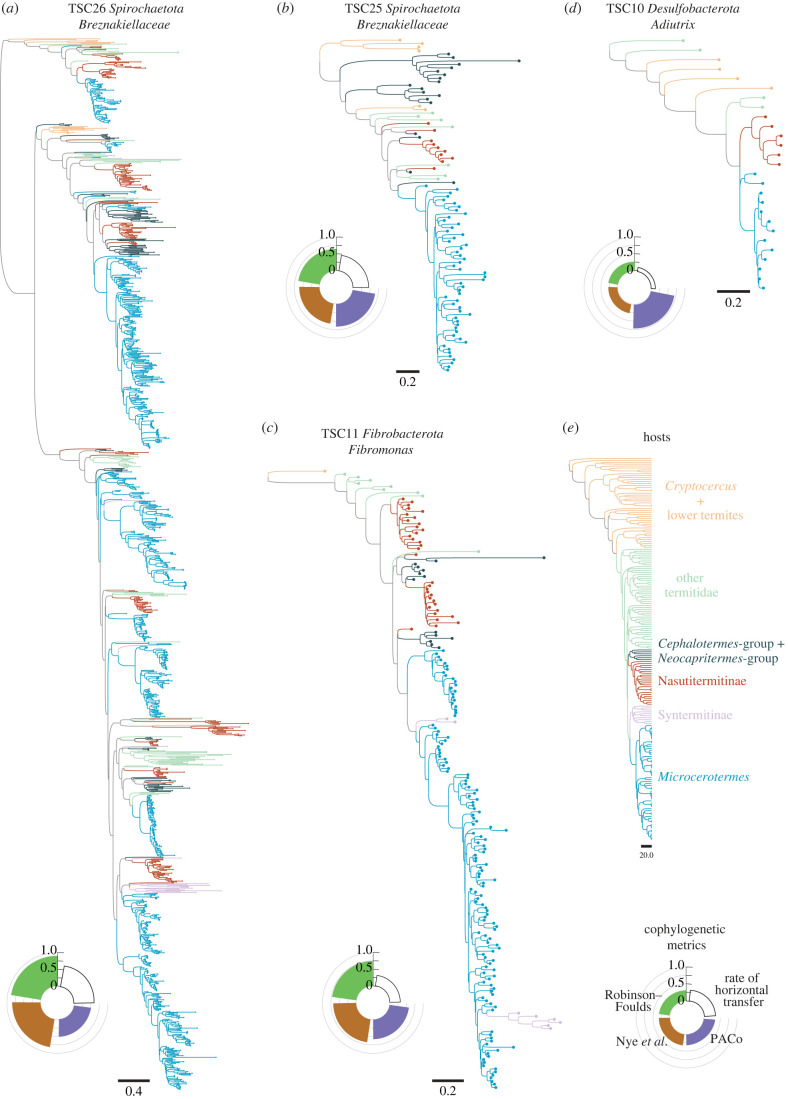
Selected phylogenetic trees of termite-specific bacterial clades (TSCs) showing strong cophylogenetic signals with termites. The TSC phylogenetic trees were reconstructed with IQtree using the RY-recoded DNA sequence alignments from the marker gene COG0552. Phylogenetic trees of (*a*) the *Spirochaetota Breznakiellaceae* TSC26, (*b*) the *Spirochaetota Breznakiellaceae* TSC25, (*c*) the *Fibrobacterota Fibromonas* TSC11 and (*d*) the *Desulfobacterota Adiutrix* TSC10. (*e*) Phylogenetic tree of termites inferred from mitochondrial genomes. The diagrams below the phylogenetic trees indicate the results of the cophylogenetic analyses and the estimation of the horizontal transfer rate. Scale bars represent substitutions per site.

In principle, the observed cophylogenetic signals between TSCs and their termite hosts could be caused by two different mechanisms: (i) vertical transmission of gut bacteria from parent colonies to daughter colonies, which is caused by the transmission of gut bacteria among family members and results in the coevolution of symbionts and hosts; (ii) limited horizontal transfers of gut bacteria among the diverging termite species due to geographical barriers, which would not require vertical transfers and results in allopatric speciation [3,4]. If vertical transfer were responsible for the cophylogenetic signals, it should give rise to bacterial lineages associated exclusively with specific termite clades and not shared with other sympatric termites. We indeed found such termite clade-specific lineages (TCSL) within many TSCs (figure 2). For example, we found several TCSLs belonging to the family *Breznakiellaceae*, the genus *Fibromonas* (phylum *Fibrobacterota*) and the genus *Adiutrix* (phylum *Desulfobacterota*) that were associated exclusively with the densely sampled genus *Microcerotermes* (figure 2*a–d*). These TCSLs were absent from the guts of other termites, including many species that are sympatric with *Microcerotermes*, demonstrating that some TCSLs are endemic to the gut of specific termite genera, as previously hypothesized based on smaller datasets [73]. They were found in the guts of *Microcerotermes* species collected across four continents and six biogeographic realms, indicating that *Microcerotermes* dispersed worldwide with their specific gut bacteria. We also found TCSLs associated with termite clades sampled less intensively. For example, a group of Nasutitermitinae that shares a common ancestor approximately 25 Mya and has been sampled across multiple continents hosted several TCSLs belonging to the family *Breznakiellaceae* and the genus *Adiutrix* (figure 2*a,b,d*). These examples of the absence of horizontal transfer of bacteria between sympatric termites belonging to different clades indicate that allopatry is not required to maintain the association between termite clades and their symbiotic bacteria. Therefore, even if allopatric speciation of termites and TCSLs likely occurred, TCSLs are transmitted vertically from parent colonies to daughter colonies and possibly horizontally among related termite species forming a clade.

**Figure 3.**
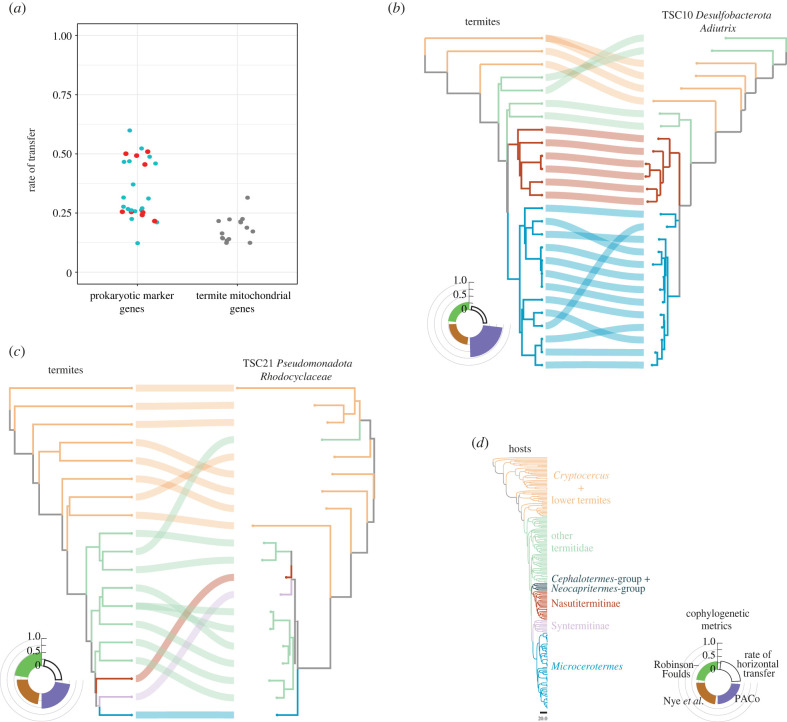
Rate of transfer and phylogenetic trees of some termite-specific bacterial clades (TSCs) showing strong cophylogenetic signals with termites. The TSC phylogenetic trees were reconstructed with IQtree using the RY-recoded DNA sequence alignments from the marker gene COG0552. (*a*) Rates of horizontal transfer of 27 TSCs were estimated using the ML method implemented in the GeneRax software. Red dots represent bacterial clades showing no significant cophylogenetic signals, and cyan dots represent bacterial clades showing significant cophylogenetic signals. Tanglegrams between termites and (*b*) the *Desulfobacterota Adiutrix* TSC10 and (*c*) the *Pseudomonadota Rhodocyclaceae* TSC21. (*d*) Phylogenetic tree of termites inferred from mitochondrial genomes. The diagrams below the phylogenetic trees indicate the results of the cophylogenetic analyses and the estimation of the horizontal transfer rate. The scale bar represents substitutions per site.

We next estimated the number of host transfer events for each TSC using the ML method implemented in the GeneRax software [69]. The estimated rates of transfer varied between 0.12 and 0.60 for TSCs showing cophylogenetic signals with termites (figure 3*a*). Note that the rates of transfer estimated with the UCE-based termite phylogenetic tree were almost identical, varying between 0.13 and 0.61 (electronic supplementary material, table S2). Notably, 16 TSCs had rates of transfer falling between 0.11 and 0.32, the range of rates of transfer estimated for each of the 13 protein-coding and two rRNA mitochondrial genes used in this study to build the phylogenetic tree of termites (figure 3*a*). Mitochondrial genes are expected to experience no transfer and have an identical evolutionary history, providing a baseline for estimated rates of transfer values obtained for genes expected to experience no horizontal transfer. While these results do not prove the absence of horizontal transfers, they suggest that the cophylogenetic patterns observed between some TSCs and termites may not involve any horizontal transfers. Cophylogenetic patterns would be obfuscated by bacterial extinction (or insufficient sequencing depth, from which it cannot be distinguished) and speciation taking place within non-speciating termite hosts [4].

Several TSCs, less speciose than *Breznakiellaceae* and *Fibromonas*, depicted patterns of cophylogeny across large parts of the termite phylogenetic tree (figure 3). For example, the phylogenetic tree of the genus *Adiutrix* found in the termite sister group Cryptocercidae, three families of termites, and across Termitidae, was highly congruent with the phylogenetic tree of termites (figure 3*b*). The phylogenetic tree of the family *Rhodocyclaceae* (phylum *Pseudomonadota*, formerly *Proteobacteria*) (figure 3*c*) is another example of a clade showing significant cophylogenetic signal with termites. We interpret these cophylogenetic patterns between termites and some of their gut bacterial symbionts as evidence of coevolution with vertical transmission taking place over several tens of millions of years.

## 4. Conclusion

We identified the oldest known cophylogenetic patterns between animals and their gut bacteria. They involve multiple bacterial lineages and their termite hosts and span tens of millions of years—some may even trace back to the first appearance of termites around 150 Ma. These findings substantiate previous claims of coevolution between termites and their gut microbiota [74] and provide concrete evidence that proctodeal trophallaxis, a social behaviour in which nest-mates exchange droplets of hindgut contents [27], indeed serves as a stable vertical transmission route over geological time scales.

## Data Availability

Raw sequence data generated in this study are available in two MGRAST projects (https://www.mg-rast.org/mgmain.html?mgpage=project&project=mgp101108 and https://www.mg-rast.org/mgmain.html?mgpage=metazen2&project=mgp84199) (see electronic supplementary material, table S1 for individual IDs). The single-copy marker gene sequences extracted in this study and the 13 phylogenetic trees of prokaryotic phyla reconstructed with COG0552 marker genes (Newick format) are available on Figshare (https://figshare.com/account/home#/projects/134324). The mitochondrial genomes sequenced in this study are available on GenBank (see electronic supplementary material, table S1). The UCE sequences are available from the Dryad Digital Repository: https://doi.org/10.5061/dryad.tmpg4f53w [75].

## Fundings

This work was supported by the Czech Science Foundation (project No. 20–20548S), the project IGA No. 20223112 from the Faculty of Tropical AgriSciences of the Czech University of Life Sciences Prague, and the Japan Society for the Promotion of Science through a DC2 graduate student fellowship awarded to J.A.
